# Supplementary material for: Basal Gp78-dependent mitophagy promotes mitochondrial health and limits mitochondrial ROS
Source: Cell Mol Life Sci. 2022 Oct 25;79(11):565. doi: 10.1007/s00018-022-04585-8 (PMC9596570; doi:10.1007/s00018-022-04585-8)
Supplement: Supplementary file 1 — Supplementary file1 (PDF 933 KB) [file 18_2022_4585_MOESM1_ESM.pdf]

**SUPPLEMENTAL FIGURE AND VIDEO LEGENDS**

**Supplemental Figure 1: Western blot analysis of mitochondrial fusion/fission and autophagy proteins in wild-type and Gp78 KO HT-1080 cells. (A)** Cell lysates from wild-type HT-1080 and 6 Gp78 KO clones were Western blotted for MFN1, MFN2, DRP1 and OPA1. Expression levels were quantified relative to corresponding β-actin blot. **(B)** Cell lysates from wild-type HT-1080 and 2 Gp78 KO clones were Western blotted for ATG5, p62, ULK1, PINK1, LC3B-I/II and quantified relative to corresponding β-actin blot. (n=3 independent biological replicates; *, $p<0.05$; ***, $p<0.001$ relative to wild-type HT-1080, if no p value indicated no significant differences were observed).

**Supplemental Figure 2: Gp78 rescues mitochondrial phenotype of Gp78 KO HT-1080 cells in a RING domain-dependent manner.** Representative maximum projections of mitochondria labelled with TOMM20 from wild type HT-1080 and Gp78 KO clones transiently transfected with wild type FLAG-Gp78, catalytically inactive RING finger (RF) mutant Gp78, or empty vector pcDNA3. Bar graph shows quantification of total mitochondrial volume per cell (n=3 independent biological replicates; 10-14 cells/condition per experiment; *, $p<0.05$; **, $p<0.01$; ***, $p<0.001$, ****, $p<0.0001$; Mean ± SEM; Scale bar: 10 μm).

**Supplemental Figure 3: The effect of variable Z-values on SPECHT spot detection.** Raw images of grey scale and merged GFP-mRFP tfLC3 of DMSO and CCCP/BafA1 treated HT-1080 cells. Corresponding Laplacian filter is presented with its resultant z-values of 1.50, 1.75, 2.0. Scale bar, 10 μm.

**Supplemental Figure 4: Impact of CCCP on MitoView 633 mitochondrial labeling. (A)** HT-1080 and Gp78 knockout clone g2-41 cells were treated with CCCP for 4 hours and labeled with the mitochondrial potential reporter MitoView 633 for 30 minutes. MitoView 633 integrated density per mitochondrial object was quantified. (n=3 independent biological replicates; >30 cells/condition per experiment; ****$p<0.001$). **(B)** Gp78 knockout clone g2-41 cells were transfected with control pcDNA plasmid, wild-type (wt) Gp78, Ring finger mutant (RM) Gp78 or the mitochondrial fission protein DRP1 and then labeled with MitoView 633. Integrated density of MitoView 633 per mitochondrial object were quantified. (Scale Bar: 10 μm; n=3 independent biological replicates; >15 cells/condition per experiment; ***$p<0.001$). **(C)** Tumor growth of HT-1080 wild-type and 6 Gp78 KO clones.

**Supplemental Figure 5:** Full Western blots for the Gp78 and β-actin blots included in Figure 1A.

**Supplemental Figure 6:** Full Western blots for the ATG5, ATPB and β-actin blots included in Figure 2A.

**Supplemental Figure 7:** Full Western blots for the LC3B-II and β-actin blots for DMSO, CCCP and starvation treated cells included in Figure 4.

**Supplemental Figure 8:** Full Western blots for the Gp78 and β-actin blots for HT-1080, HeLa, PC3 and PANC1 cells included in Figure 7.

**Supplemental Video 1**: Time lapse movie of GFP-mRFP-positive tfLC3 puncta (yellow) and MitoView 633 labeled mitochondria in an HT-1080 cell (blue SPECHT outline overlaid on raw MitoView 633 image). Images acquired every 10 seconds.

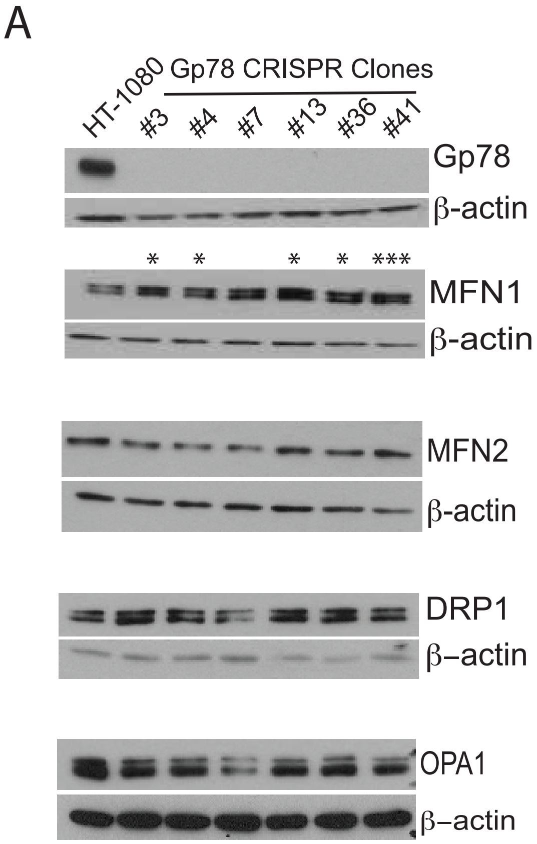


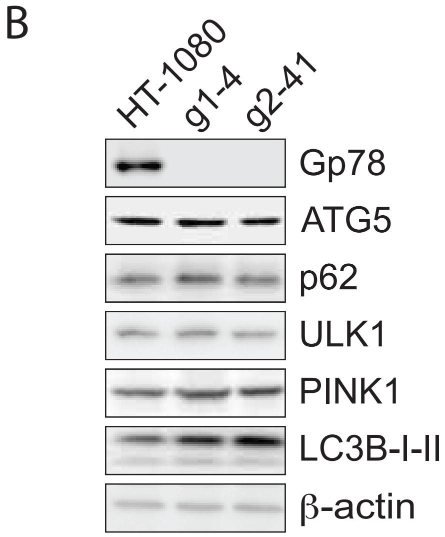


Supplemental Figure 1

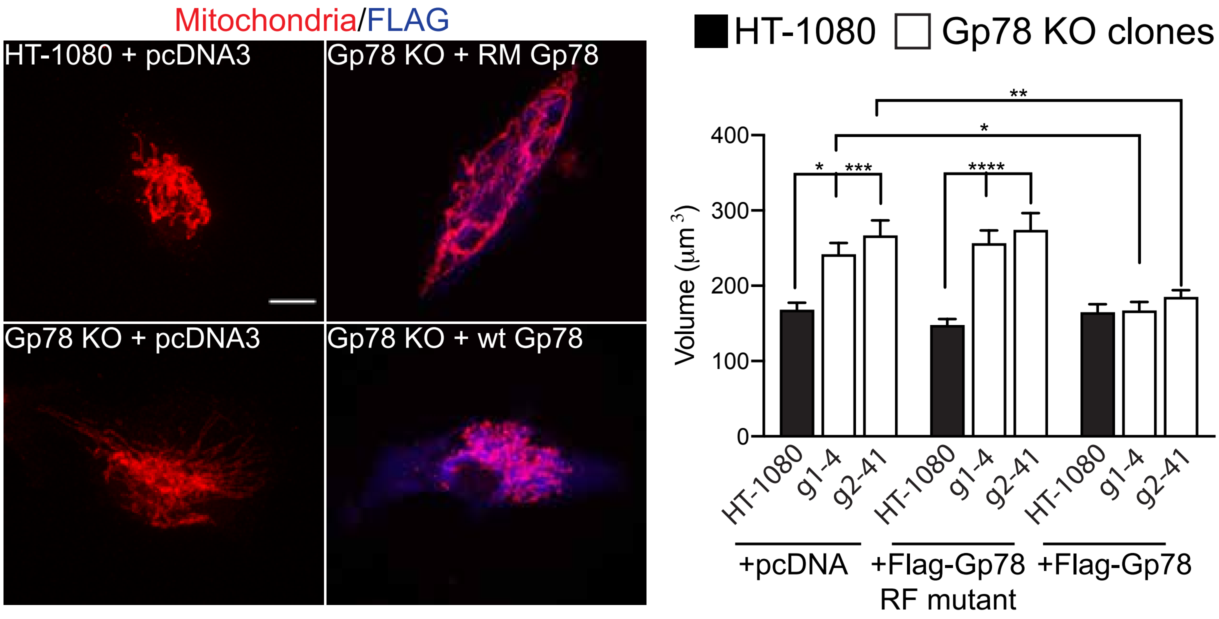


Supplemental Figure 2

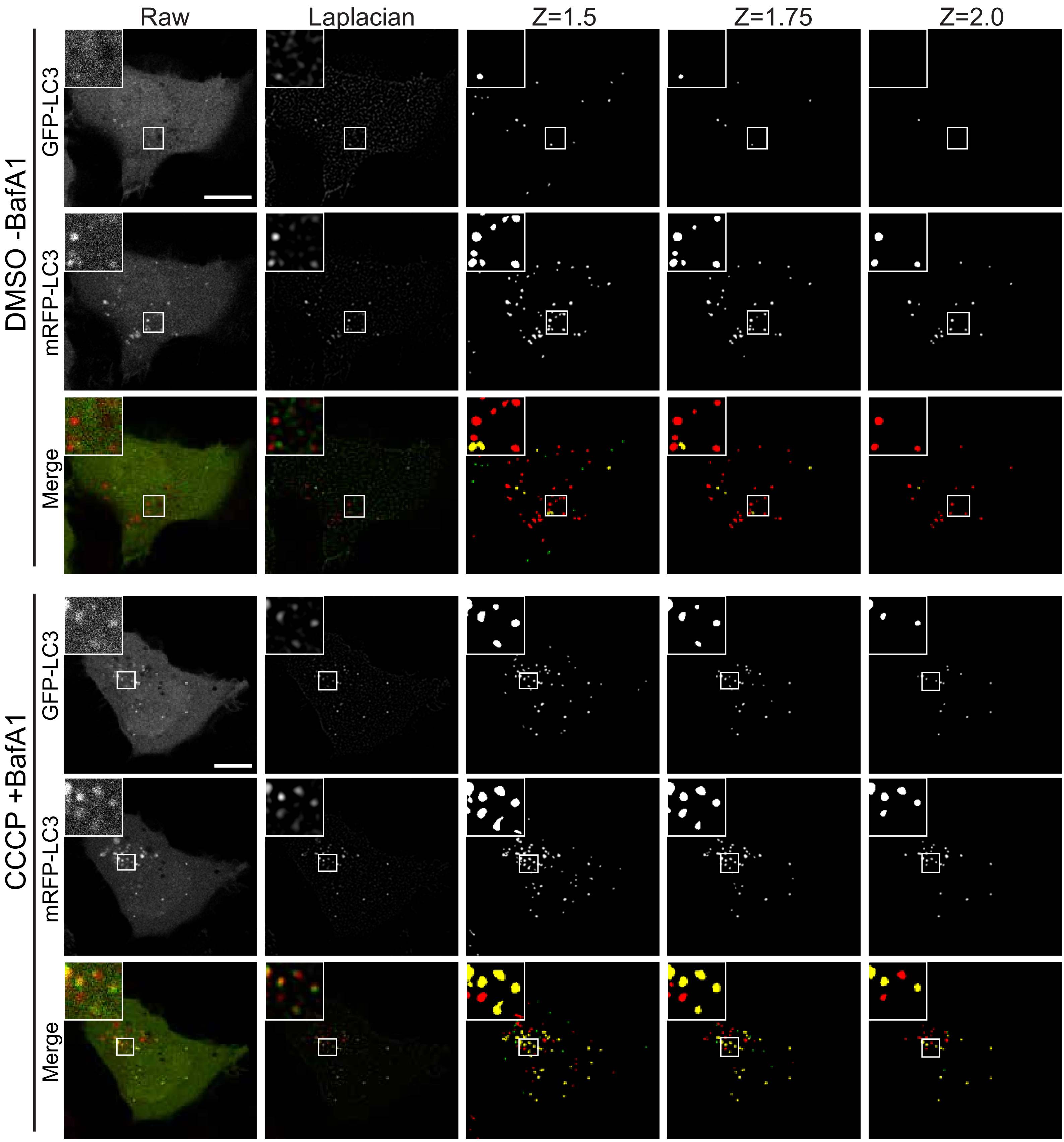


Supplementary Figure 3

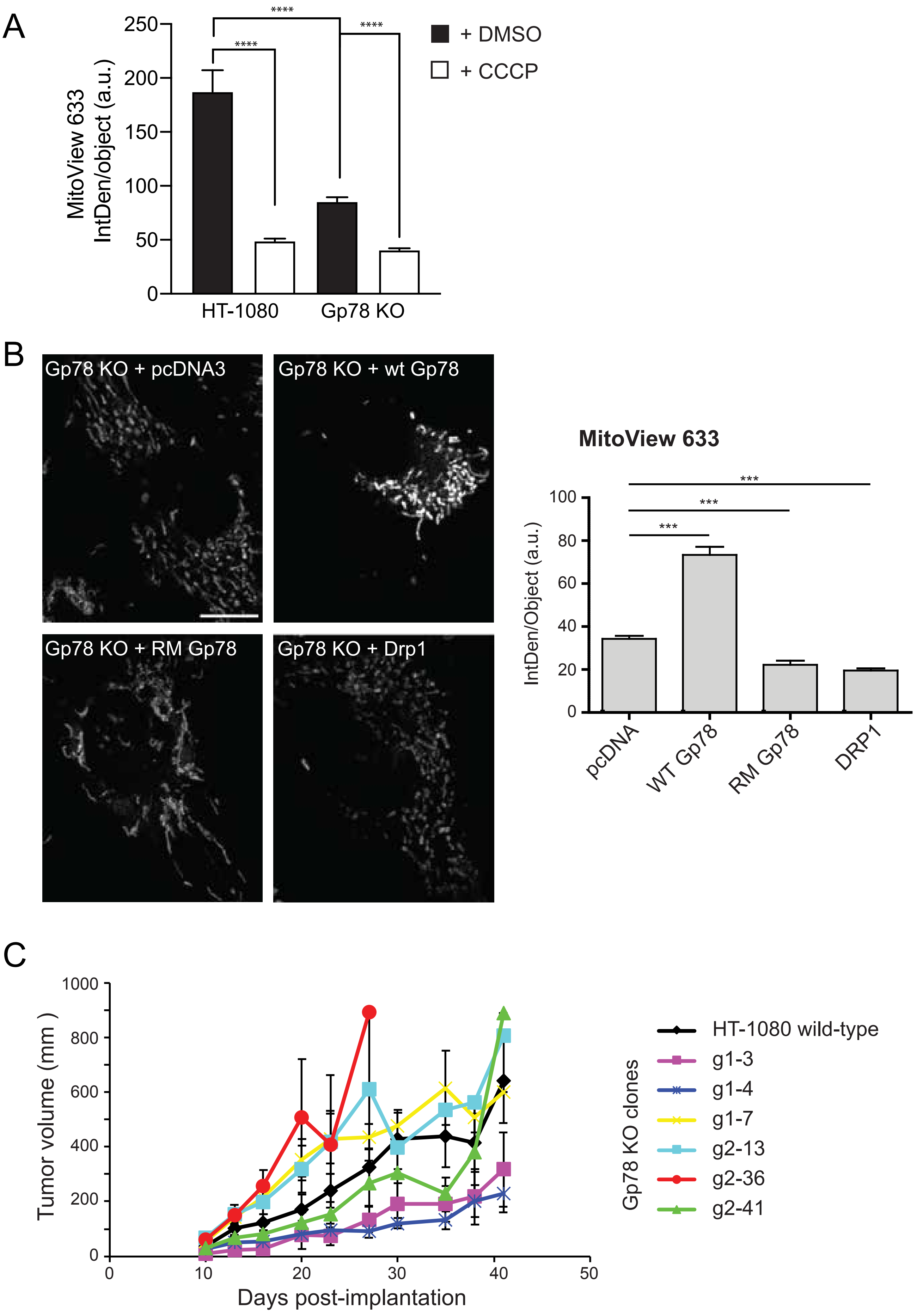


**Supplemental Figure 4**

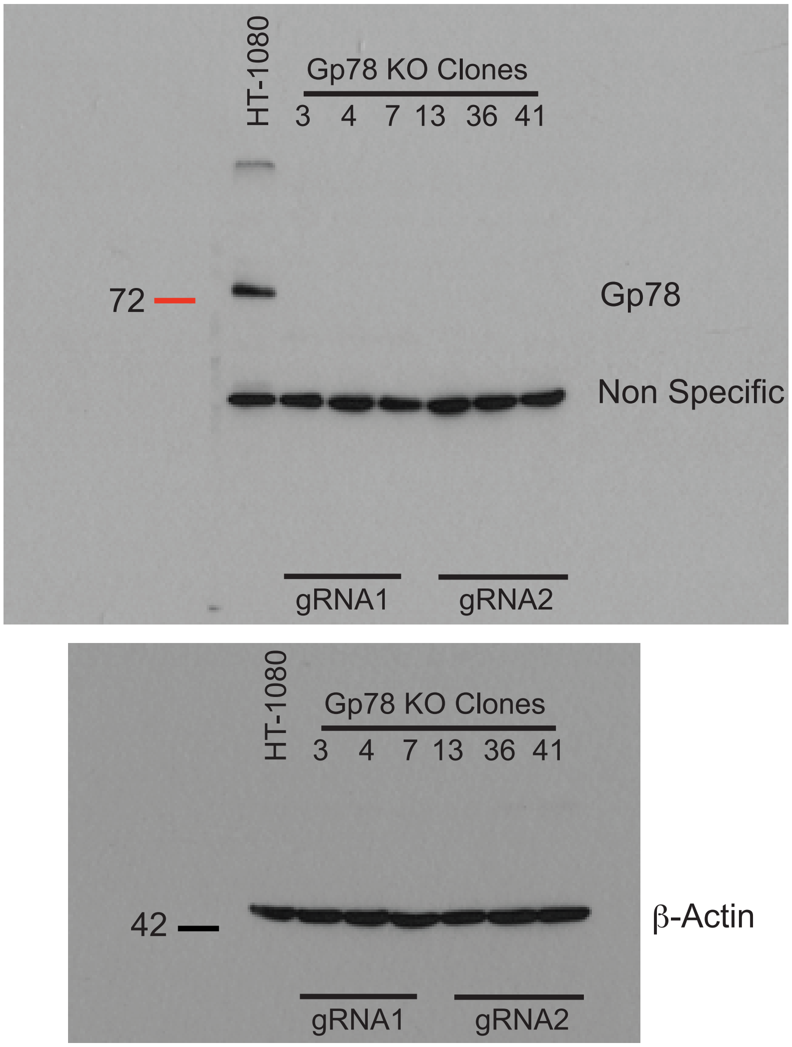


**Supplemental Figure 5 (Full blots for Figure 1A)**

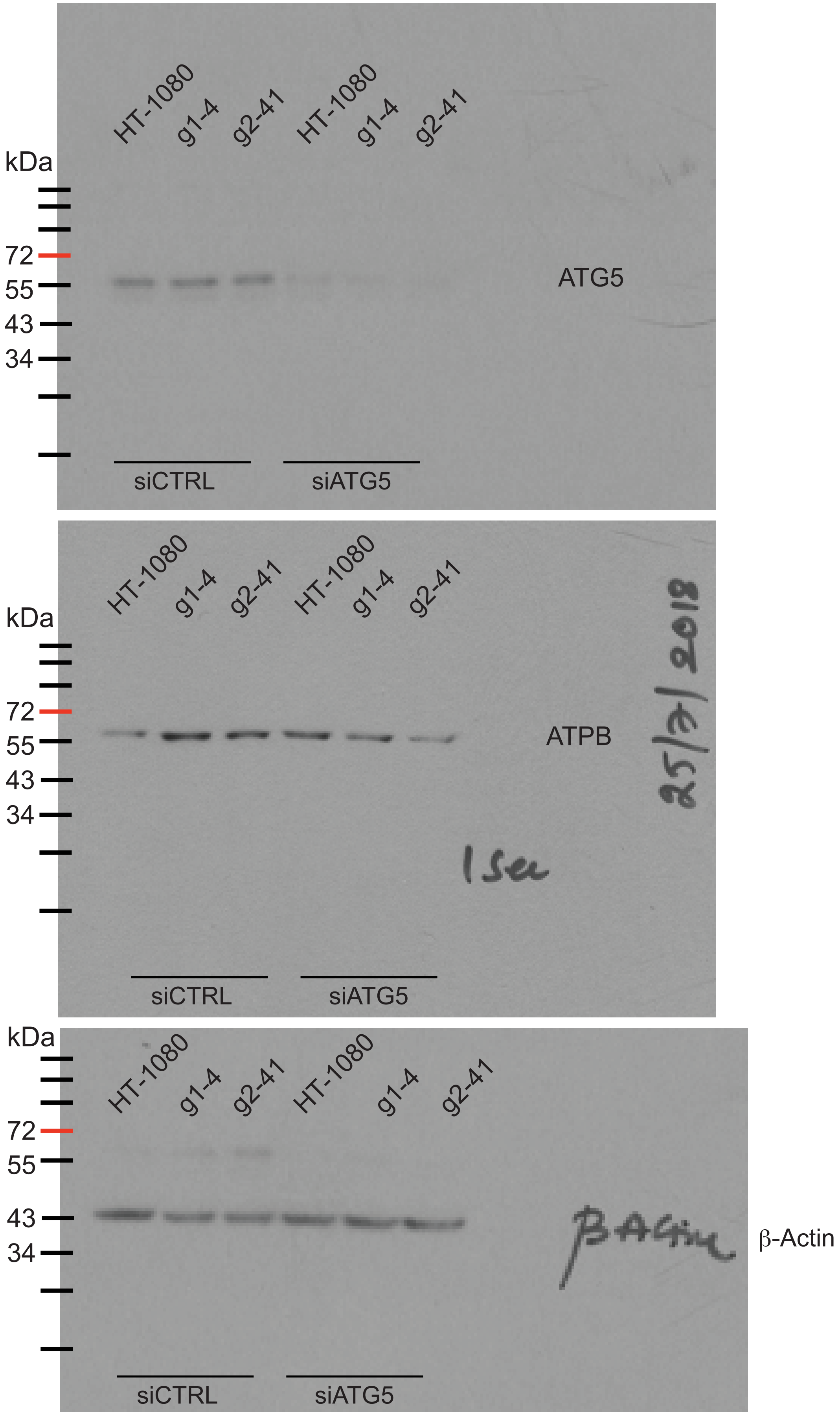


**Supplemental Figure 6 (Full blots for Figure 2A)**

## FIGURE 3-DMSO-CCCP

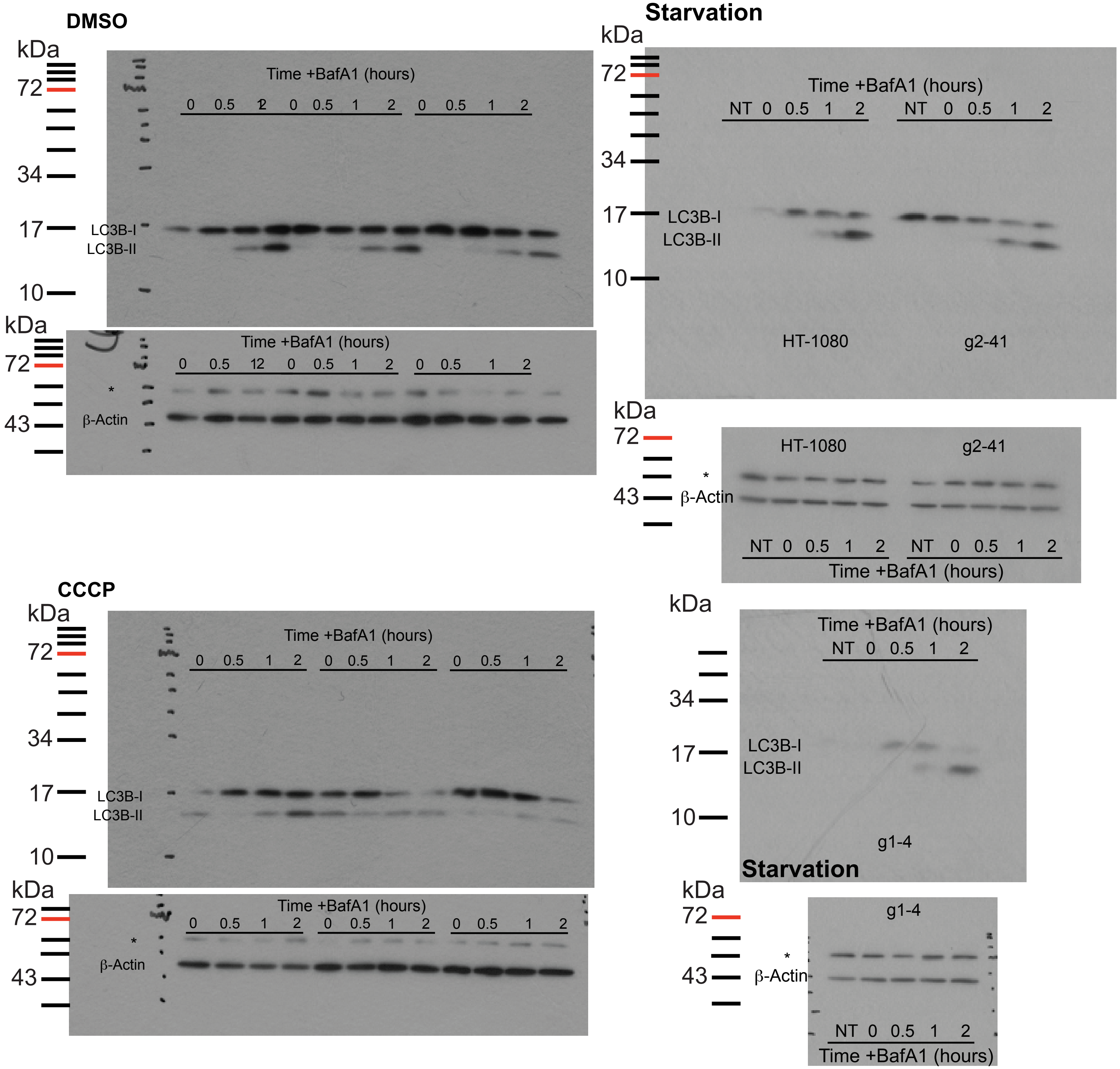


**Supplemental Figure 7 (Full blots for Figure 4)**

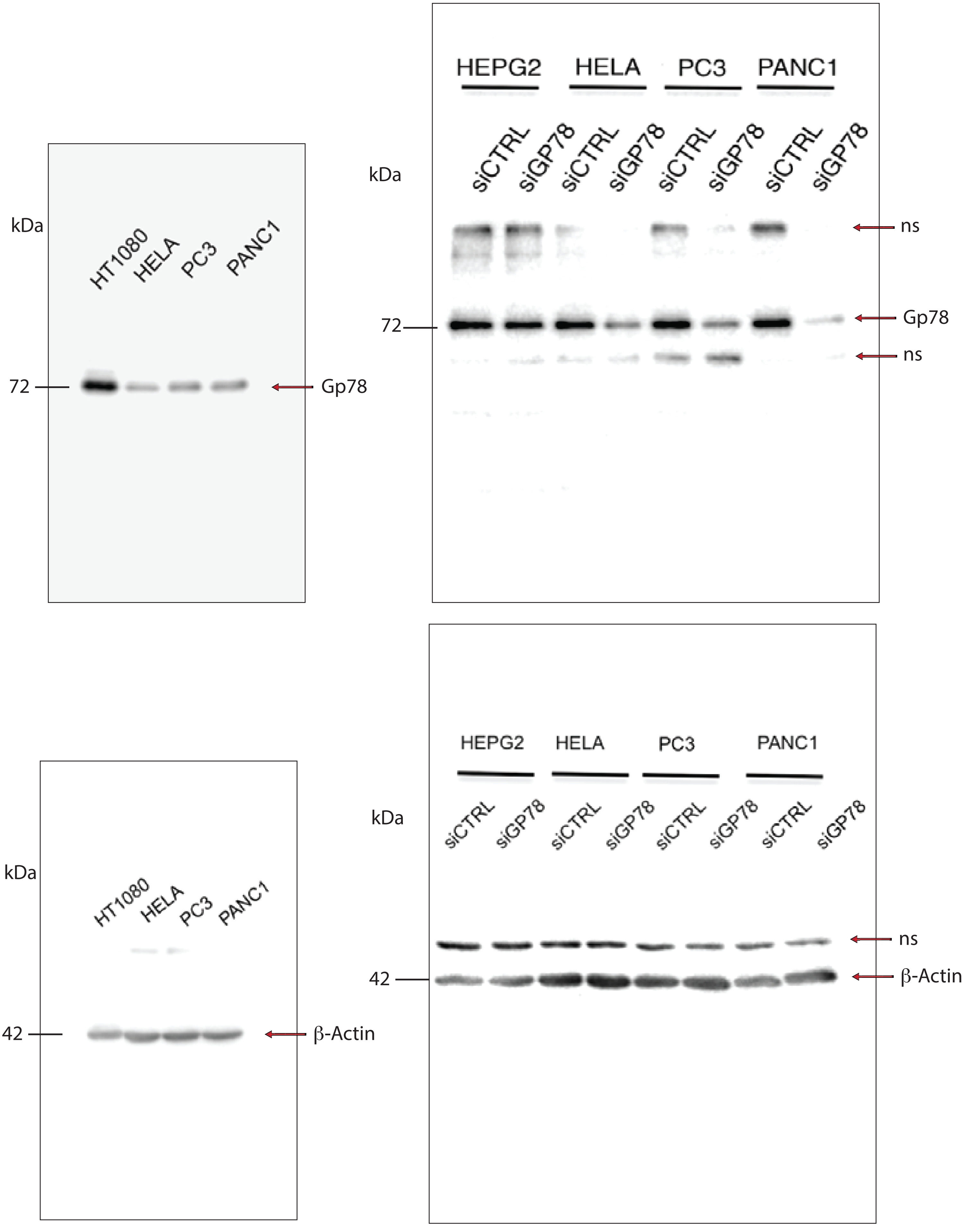


**Supplemental Figure 8 (Full blots for Figure 7)**
